# Analectic

**Published:** 1828-04

**Authors:** 


### MISCELLANEOUS INTELLIGENCE.

---

### I. ANALECTIC.

1. *On Amputation and the Omission of Ligatures to the Vessels.* By Dr. L. Koch, of Munich. Notwithstanding the numerous researches and experiments on the alterations and condition of compressed vessels at the place of ligature, it is surprising that the most inaccurate ideas are still entertained on the subject, and that the opinions of physicians and surgeons, on the spontaneous cessation of hæmorrhage from divided vessels, should still be so contradictory and uncertain. Dr. Koch thinks that the subject has not been studied minutely enough, or the phenomena observed with sufficient impartiality to arrive at the truth. He believes that timidity also has prevented our observing that the ligature is useless in many cases, while experience has shown its disadvantages. There are few who will amputate a limb, and fearlessly trust to nature for the security of the cut vessels. Dr. K's father, director of the General Hospital of Munich, "*has not tied a single artery in the various amputations which he has performed for the last twenty years.* To this wide range of experience, the son has added his own, in corroboration of the opinions of his father and of himself respecting the imaginary danger of leaving vessels untied in amputations.

Arteries, says he, when cut and not tied, remain entirely open, up to the place where they are divided:—The canal of arteries tied in the usual manner remains open also to the spot where the ligature is applied, and their parietes do not unite at this spot. These observations were repeatedly made by the author's father on dead bodies, where the arteries had been cut by him, or tied by other surgeons many years previously. He always found the diameter of the vessels that had *not* been tied, contracted as they approached the place of section, but the parietes never adherent till the artery ended in a kind of cicatrix. These things are seen in numerous preparations by the author, in the anatomical museum of Munich. The vessels that had been tied presented the same appearances, except that, at the spot where the thread had been applied, there was a narrowing, but never an obliteration of the canal of the vessel.

In a disarticulation of the hand, the surgeon had tied the radial artery, and omitted to tie the ulnar, as it did not bleed. The ligature came away on the 8th day, and on the succeeding day the patient died. On examination, the terminations of the two arteries were so similar, that it was difficult to say which of them had been tied by ligature. Both extremeties were perfectly pervious—the radial artery appeared to be slightly torn at the termination.

In numerous experiments on dogs, our author could perceive no difference between the arteries that had been tied, and those that were left to nature. Thus, he tied the femoral artery of a dog, and cut the vessel below the ligature, without hæmorrhage. The wound was closed and healed. A month afterwards he killed the animal, and found the upper and lower extremities of the vessel were completely similar, each being united to an external coagulum by an open mouth.

The formation of a coagulum takes place in some, but not in all cases. But it produces the same effects in the vessels which are tied, and in those which are cut, and not tied. In ligatures of arteries the internal coagulum is often found in connexion with the external, so as to fill exatly the orifice of the vessel. The coagulum is rarely adherent to the internal parietes of the vessel, and never completely so up to the nearest anastomosis. An amputation was performed at the hip-joint, and the crural artery was tied. The ligature came away on the eleventh day, and, on the 14th day, the patient died. On dissection, a clot was found plugging the artery, but the canal of the vessel was open. In a very few cases indeed was the bore of the artery found obliterated after the ligature, by adhesion of the sides of the vessel.

No doubt, says the author, "that most surgeons will stare when I propose the general abandonment of the ligature, as the means of preventing hæmorrhage, especially in amputations. They will be still more surprised when I assert, that by this omission of the ligature, the most certain means are taken to obviate effusion of blood. Yet this assertion rests on the basis of experience, and can be testified to by all those who have witnessed my father's operations in a public hospital for twenty years past."

Dr. K. appears to think that the spontaneous cessation of hæmorrhage from a divided vessel, depends *chiefly* on some change in the blood itself—partly in retraction of the vessel. The coagulum he considers as the *effect* rather than the *cause* of this cessation of hæmorrhage. This last conclusion appears plausible; for it is hard to conceive that coagulum can form *during* hæmorrhage—and if it form after the cessation of the flow of blood through the orifice of the vessel, it can hardly be viewed in the light of a *cause* of that cessation. All that can be said in this case is, that the coagulum may prevent subsequent hæmorrhage; but this our author denies.

"The application of the ligature," says he, "in disturbing the spontaneous cessation of the hæmorrhage, acts in a manner quite opposed to the end in view. It produces, it is true, a mechanical and temporary obliteration of the bore of the artery, but this is inferior in value to the natural retraction of the vessel, and spontaneous cessation of the hæmorrhage."

This spontaneous cessation is to be aided, or rather promoted, by pressure on the trunk of the vessel leading to the part, and a gentle degree of the same on the face of the stump, either by the hand or by a proper bandage. By these means the stasis of the blood is promoted, and protection from future hæmorrhage secured.

The method pursued by Dr. K. and his father in amputations is as follows:—After dividing the soft parts and bone, the surface is sponged, and the muscles and integuments brought neatly into contact, and retained by adhesive plaster, so as to secure adhesion by the first intention, if possible. *During* the operation, the vessel is compressed by the fingers of an assistant, and *afterwards* the pressure of the fingers is rendered unnecessary by the application of a compress, laid along the trajet of the main artery, secured by a roller. The patient is then placed in his bed, and the stump kept elevated, and an assistant is directed to make gentle pressure on the face of the stump, for an hour or two—or longer, if he feel considerable pulsation in the part. "When this pulsation has ceased, and when the dressings appear tinged red by the exuding lymph, all danger of hæmorrhage is considered as at an end, provided the patient keeps quiet. Presently, the exudation of lymph ceases—and the dressings become quite dry and cold." The patient generally passes the first few days without fever, on which account he is allowed wine, coffee, and other food, which dare not be given under other circumstances. No opiates or medicines of any kind are usually exhibited after the operation. About the fifth day, there is generally some traumatic pyrexia evinced, owing to the suppurative process going forward in the wound; but it requires no particular treatment. A moisture taking place on the dressings about the seventh day, indicates the establishment of suppuration: but if the dressings keep dry, union by the first intention is sure to have occurred. Whether suppuration or adhesion has taken place, the dressings are never removed before the tenth day, or even later, unless violent inflammation or hæmorrhage should arise. They consider that the adhesion of the integuments and muscles is never properly cousolidated before the tenth or twelfth day, and therefore that much mischief is done by too early a removal of the dressings."—*Journal des Progres, from the Journal fur Chirurgie und Augenheilkundie von Græfe and Walther, p. 9. t. 560.—Am. Journ. of the Med. Sciences, No. 2.*

2. *Chorea.*—M. Lisfranc presented to the Royal Academy of Medicine at their sitting of the 16th of August last, a lady whom he had cured of chorea by general bleedings, and repeated application of leeches to the upper part of the spine: he had been induced to try this mode of treatment, because M. Serres had assured him that he almost always found the tubercula quadrigemina in a state of inflammation in the bodies of those persons who died of chorea. M. Serres, who was present, stated that in four cases of chorea he found the tubercula quadrigemina altered; in one case a fatty tumour was developed on them: in another, there were marks of considerable excitement, with bloody effusion at the base of these bodies. In the two last cases, the whole substance of the corpora quadrigemina was inflamed, and the inflammation extended to the roof of the fourth venticle. The symptoms appeared to him to have some relation with an injury of this part of the brain. M. Serres tried experiments on living animals, and found that those animals in which this part of the brain was injured, had motions similar to those observed in cases of chorea. M. Rolando had also perceived this fact in his experiments. M. Serres was not, however, willing to conclude that in every case of chorea the tubercula quadrigemina are injured; he had seen several in which no injury in the brain could be discovered. As persons afflicted with this disease generally experience great pain at the back part of the head, above the region of the neck; he had been induced to apply remedies to this spot, and which had often cured the complaint in its acute state; but when it becomes chronic, the frequent application of leeches, in the neighbourhood of the supposed seat of the disease, prove of no benefit.—*Archives Generales de Medecine, Sept.* 1827.—*Ibid.*

3. *On the distinction between Rheumatic Inflammation of the Heart and Inflammation of the Serous Membrane of the Pericardium.* By Robert Adams, Esq.—The distinction between rheumatism of the heart and of its serous membrane, has not been sufficiently insisted upon, and as it is a matter of consequence in practice to be able to distinguish them, we copy from Mr. Adams' excellent paper on the diseases of the heart, in the fourth volume of the *Dublin Hospital Reports*, the diagnostic characters of these two affections, which he has laid down with great accuracy and precision.

"In the one case the organ is simply, and often but transiently affected, just as any other muscle is, the person has perhaps been affected with rheumatic pains in the loins, with but little fever; these suddenly leaving this region run to the diaphragm, and cause a temporary affection of the breathing, with what the patient calls spasms in the chest. The countenance undergoes sudden changes: there are at such moments strong beats of the heart, and intermissions of the pulse sensible to the patient; and in females I have sometimes seen such attacks end in an hysteric paroxism, and all symptoms subside when the lumbar pains returned. In such cases, the tongue is somewhat foul, the skin is frequently relaxed by profuse perspiration, and the urine is remarkably turbid; but the pulse has neither the frequency, hardness, nor peculiar vibratory feel that it has in the other, and more dangerous case: the countenance does not betray that anxiety, or, as it is denominated by some authors, that anguish which it almost uniformly expresses when the membranes of the heart are affected with acute inflammation, from whatsoever cause proceeding. Although simple rheumatism of the musculur structure of the heart may occasionally pass on to carditis, or to an inflammatory affection of the serous membrane of the heart, there cannot be a doubt that the two cases are very distinct from each other, and require treatment so different as to make it highly important for the practitioner to be aware of the risk of mistaking the comparatively simple case of rheumatism of the heart, for the far more urgent and dangerous one of rheumatic inflammation of its serous membrane. The latter disease I have usually seen in children, and persons about, and under the age of puberty, in whom, metastasis seems much more liable to occur, than in those more advanced in life; and the sudden translation of the rheumatic inflammation to the heart has usually occurred where the synovial system of the extremities was the original seat of the disease: when the muscles have been affected with acute rheumatism, metastasis of the heart has not, to my knowledge, been frequent, although I have seen the diaphragm affected by it in such a manner as to excite apprehensions for a time, that the former more important organ was implicated. There seems to be a greater disposition in the acute inflammation to pass from the synovial membranes to the serous, than to any other tissues; which is not so much to be wondered at, when their great similarity of structure, appearances, and functions, is considered.—*Ibid.*

